# Neonatal and early infancy antibiotic exposure is associated with childhood atopic dermatitis, wheeze and asthma

**DOI:** 10.1007/s00431-024-05775-1

**Published:** 2024-09-28

**Authors:** Santeri Räty, Helena Ollila, Olli Turta, Anna Pärtty, Ville Peltola, Hanna Lagström, Johanna Lempainen, Samuli Rautava

**Affiliations:** 1 Department of Paediatrics, University of Turku, Kiinamyllynkatu 4-8, 20520 Turku, Finland; 2 Department of Biostatistics, University of Turku and Turku University Hospital, Turku, Finland; 3 Department of Paediatrics and Adolescent Medicine, Turku University Hospital, Turku, Finland; 4 Department of Public Health, University of Turku, Turku, Finland; 5 Centre for Population Health Research, University of Turku and Turku University Hospital, Turku, Finland; 6 Immunogenetics Laboratory, Institute of Biomedicine, University of Turku and Clinical Microbiology, Turku University Hospital, Turku, Finland; 7 Department of Pediatrics, University of Helsinki, Helsinki, Finland; 8 New Children’s Hospital, Helsinki University Hospital, Helsinki, Finland

**Keywords:** Antibiotics, Asthma, Atopic, Dermatitis, Newborn

## Abstract

Antibiotics are frequently administered in the neonatal period and early infancy. Little is known about the long-term health consequences of early life antibiotic exposure. The objective is to investigate the association between neonatal and early life (0–6 months) antibiotic treatment and the development of atopic dermatitis, asthma and the use of inhaled corticosteroid medication later in childhood. We analyzed data obtained from hospital records and national registers in a cohort of 11,255 children. The association between early antibiotic exposure and the outcomes were analyzed using logistic regression. Confounding factors were included in the model. Neonatal antibiotic therapy for confirmed infection was associated with childhood atopic dermatitis (adjusted odds ratio 1.49; 95% confidence interval 1.15–1.94). Antibiotic therapy by six months of age was more common in children developing atopic dermatitis (adjusted odds ratio 1.38; 95% confidence interval 1.15–1.64), asthma (adjusted odds ratio 1.56; 95% confidence interval 1.32- 1.85) and inhaled corticosteroid medication use (adjusted odds ratio 1.88; 95% confidence interval 1.66–2.13).

*Conclusions*: Neonatal antibiotic therapy for confirmed or clinically diagnosed infection is associated with increased risk of atopic dermatitis later in childhood. Antibiotic treatment before six months of age is associated with atopic dermatitis, asthma and inhaled corticosteroid use.

**Table:** 

**What is known:** *• The use of antibiotics early in life has been associated with an increased risk of developing atopic dermatitis or asthma.* *• Confounding by indication or reverse causation may underlie the observed associations.*	
**What is new:** *• Our results demonstrate that neonatal antibiotic therapy for confirmed or clinically diagnosed infection was associated with increased risk of atopic dermatitis and antibiotic treatment before six months of age was associated with atopic dermatitis, asthma and inhaled corticosteroid use in analyses adjusted for confounding factors.*

**Supplementary information:**

The online version contains supplementary material available at 10.1007/s00431-024-05775-1.

## Introduction

Antibiotics are among the most frequently used medications in children. In the United States, intravenous antibiotics are administered to 2–42% of all neonates [1] and by the age of two years, children have on average received nearly three courses of antibiotics [2]. While antibiotics are vitally important, antibiotic exposure in early life may also have detrimental consequences [3].

Epidemiological studies have suggested increased risk of developing atopic dermatitis and asthma in children exposed to antibiotics during the first months or years of life [4–8]. However, observational studies are prone to confounding by indication since early-life respiratory tract infections have been implicated in the pathogenesis of asthma [9]. Children developing asthma may also be more likely to manifest with more severe infections of the respiratory tract and be therefore treated with antibiotics. Newborns subjected to antibiotic therapy provide an opportunity to investigate the impact of antibiotic exposure with little risk of reverse causation or confounding by indication.

We have recently reported that antibiotic administration in the neonatal period and in early infancy may have age-dependent associations with later growth and BMI [10]. Antibiotic treatment in the first week of life has been observed to be associated with parentally reported childhood asthma in one large cohort study [11]. We hypothesised that antibiotic exposure in the neonatal period or during the first six months of life is associated with increased occurrence of atopic dermatitis, asthma or the use of inhaled corticosteroid medication later in childhood in an unselected, population-based, prospective birth cohort.

## Materials and methods

### Study subjects

**Fig. 1:**
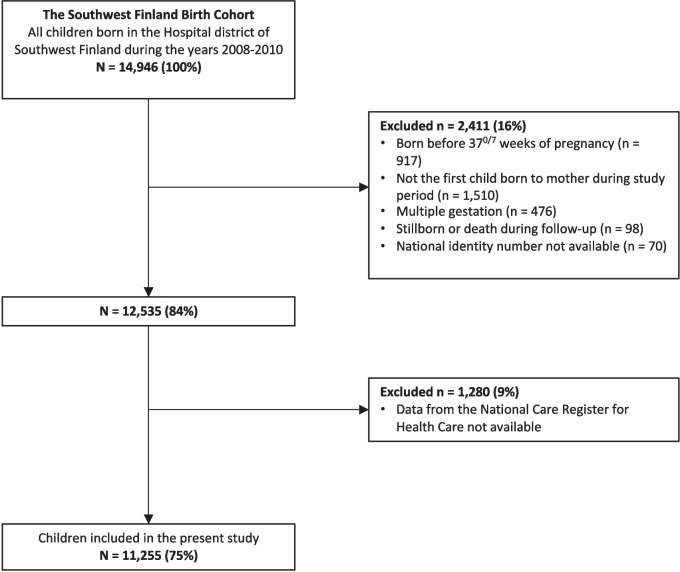
Flowchart summarizing the subjects included in the study from the Southwest Finland Birth Cohort. The number of subjects excluded at the first stage is less than the sum of the individual numbers because an individual subject may have belonged to several exclusion categories

The study is based on the prospective Southwest Finland Birth Cohort (SFBC), which consists of all 14,946 children born in the hospital district of Southwest Finland during the years 2008–2010. Subjects born from singleton pregnancies after 36^6/7^ weeks of pregnancy were included in the present study. If a woman had more than one pregnancy and delivery during the study period, only the first child was included in this study to ensure the independence of the study subjects. Subjects with incomplete identifier data were excluded from the study. Altogether 11,255 children were included in the present study (Fig. 1).

Data regarding neonatal antibiotic exposure and diagnoses of neonatal bacterial infections during the first 14 days of life were extracted from the hospital records [10]. As per the hospital policy, initial antibiotic therapy in the neonate consisted of a combination of intravenous benzylpenicillin and gentamicin. The children were grouped according to neonatal antibiotic exposure as follows: 1) no neonatal antibiotic exposure, 2) empirical antibiotic therapy, which was discontinued after infection had been ruled out, or 3) antibiotic therapy for confirmed or clinically diagnosed infection. Data on antibiotic purchases during the first six months of life was extracted from the Drug Prescription Register maintained by the Social Insurance Institution of Finland.

Data regarding previous deliveries, maternal prepregnancy body mass index (BMI), smoking during pregnancy, duration of pregnancy, mode of delivery, child’s sex, birth weight, intrapartum antibiotic treatment and laboratory investigations during the first 7 days of life were extracted from the hospital records and national registers. The diagnoses of atopic dermatitis (ICD-10 code L20.0) and asthma (ICD-10 codes J45 and J46) were obtained from the National Care Register for Health Care maintained by the Finnish Institute for Health and Welfare, which covers all visits at public hospitals in Finland. The median (Interquartile range (IQR)) follow-up time for the children in the cohort was 8.1 years (7.7, 9.3) for atopic dermatitis, 8.2 years (7.7, 9.3) for asthma and 8.9 years (8.8, 10.6) for inhaled corticosteroid medication. According to the national guideline [12] for the diagnosis and treatment of asthma at the time of the study, the diagnosis of asthma in children younger than three years was based on asthma-like symptoms that occur more than twice a week that can be relieved with inhaled bronchodilator medication. Alternatively, asthma may be diagnosed in a child with risk factors for asthma, recurrent symptoms and doctor-diagnosed wheezing at least three times during a period of one year. In children older than three years asthma is preferably diagnosed based on measurements of respiratory function.

Data on inhaled corticosteroid use was recorded to objectively reflect doctor-diagnosed childhood wheezing and asthma-like symptoms. Purchases of inhaled corticosteroids were extracted from the Drug Prescription Register maintained by the Social Insurance Institution of Finland.

Subjects with missing data regarding previous deliveries, maternal prepregnancy body mass index (BMI), smoking during pregnancy, duration of pregnancy, mode of delivery, child’s sex, birth weight and intrapartum antibiotic treatment were not included in the statistical models. Altogether 81 (0.7%) subjects were omitted due to missing values for the response or explanatory variables in the analyses pertaining to atopic dermatitis. The respective number was 473 (4.2%) in the case of asthma and 81 (0.7%) in the case of inhaled corticosteroid medication use.

### Statistical analyses

Atopic dermatitis, asthma, inhaled corticosteroid medication use and asthma together with inhaled corticosteroid medication use were selected as the response variables. Neonatal antibiotic exposure and antibiotic use after the neonatal period but during the first 6 months of age (classified as yes vs no) were the main explanatory variables. Only antibiotic exposure prior to diagnosis was considered. The following potential confounding factors were included in the statistical analyses: having older siblings (previous pregnancies yes vs no), maternal BMI before pregnancy, smoking during pregnancy, duration of pregnancy, mode of delivery (vaginal vs caesarean section delivery), child’s sex and intrapartum antibiotic exposure. Gestational age and maternal BMI before pregnancy were treated as continuous variables and the remaining variables as categorical variables. The statistical analysis was first performed using univariate analysis to explore relationships between the explanatory variables and the response variables (t-test for continuous variables and the chi-squared test for categorical variables). The data are presented as means with 95% confidence interval (CI) for continuous variables and percentage with counts for categorical variables. Sensitivity analysis was performed for maternal BMI before pregnancy with Wilcoxon rank-sum tests because of non-normal distribution. Associations between the response variables and maximum plasma C-reactive protein (CRP) concentration during the first week of life were examined with Wilcoxon rank-sum test. The chi-squared test was used for categorical plasma CRP concentrations.

**Fig. 2:**
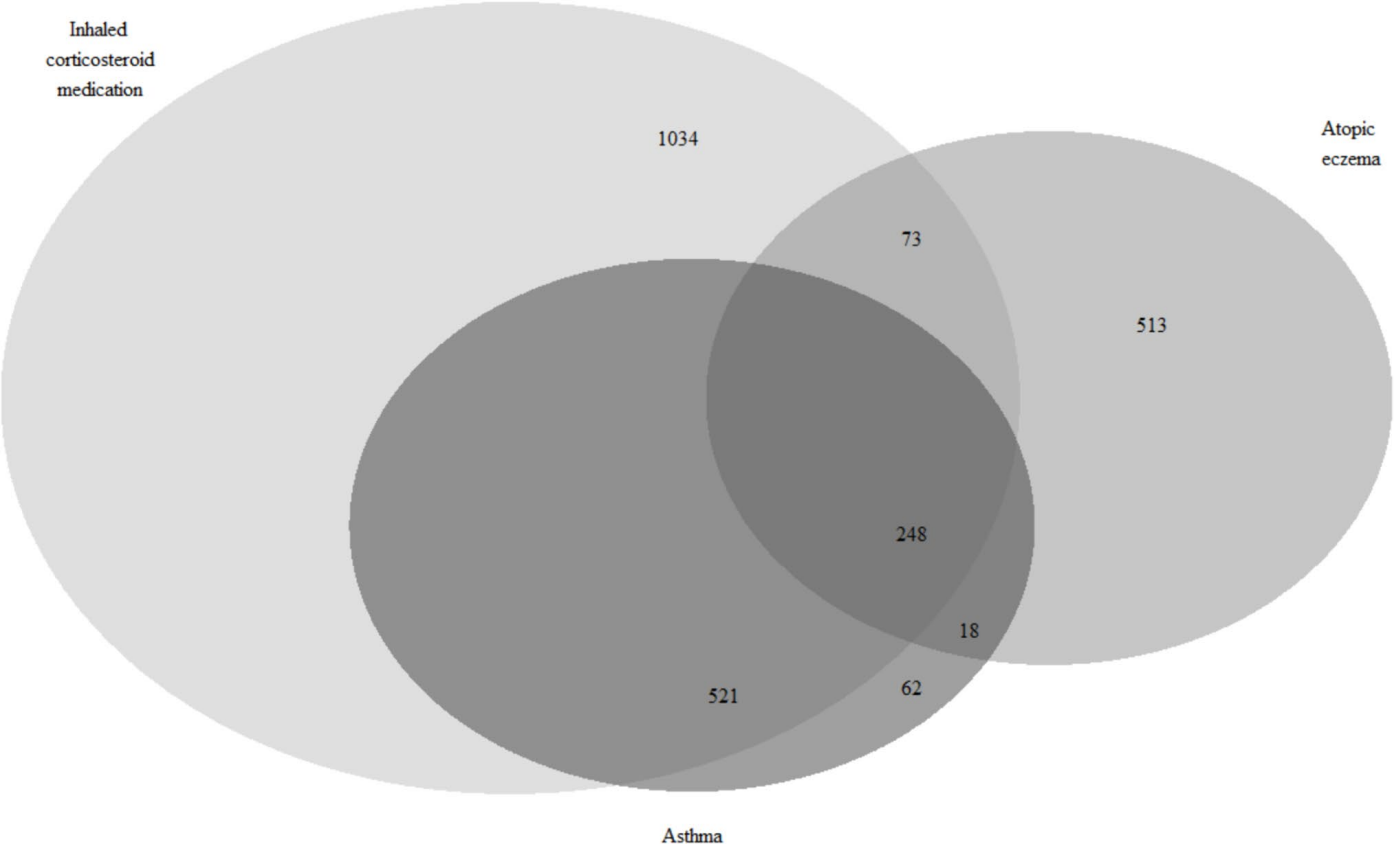
Venn diagram depicting the subjects diagnosed with atopic dermatitis and asthma or using inhaled corticosteroid medication in the study cohort of 11,255 children

Atopic dermatitis, asthma and inhaled corticosteroid medication use were subsequently analyzed using logistic regression with the “LOGISTIC” procedure of SAS. Neonatal and infant antibiotic exposure, older siblings, maternal prepregnancy BMI, smoking during pregnancy, duration of pregnancy, mode of delivery, child’s sex and intrapartum antibiotic treatment were included in the model as explanatory variables. The level of significance was set at *p* < 0.05. Analyses and odds’ ratio (OR) figures were conducted with the SAS software, version 9.4 for Windows (SAS Institute Inc., Cary, NC, USA). The Venn diagram (Fig. 2) was constructed in R version 3.6.1 using the VennDiagram package.

### Ethical approval and trial registration statement

The study was conducted in accordance with the Declaration of Helsinki. The study was approved and found ethically acceptable by the Finnish National Institute for Health and Welfare. The legal basis for processing of personal data is public interest and scientific research (EU General Data Protection Regulation 2016/679 (GDPR), Article 6(1)(e) and Article 9(2)(j); Data Protection Act, Sections 4 and 6).

## Results

Detailed clinical characteristics of the 11,255 children in the study cohort are presented in Table 1. Altogether 1,255 (11.2%) children were exposed to antibiotics during the first 14 days of life. Empirical antibiotic therapy was administered to 592 (5.3%) subjects in whom infection was subsequently ruled out (median duration of antibiotic therapy 2 days, IQR 2, 2). Neonatal infection was confirmed or clinically diagnosed in 663 (5.9%) neonates (median duration of antibiotic therapy 7 days, IQR 7, 7). Altogether 1,777 (15.8%) children had received antibiotic treatment after the neonatal period but before 6 months of age. Plasma CRP concentration was measured during the first week of life from 1,331 (11.8%) of the study subjects. The characteristics of the subjects exposed and not exposed to antibiotics are presented in Supplementary Table 1.

**Table 1:** Clinical characteristics of the children developing atopic dermatitis (a), asthma (b) or using inhaled corticosteroid medication (c) in the study cohort

a)					
	**N (%)**	**Total**	**Atopic dermatitis**	**No atopic dermatitis**	**P**
		*n* = 11,255	*n* = 852	*n* = 10,403	
**Maternal characteristics**					
Previous births, No. (%)	11,255 (100)	5,821 (52)	473 (56)	5,348 (51)	0.021
Maternal prepregnancy BMI *, median (CI)	11,198 (99)	23.4 (23.3, 23.4)	23.2 (22.9, 23.5)	23.4 (23.3, 23.5)	0.31
Smoking during pregnancy, No. (%)	11,227 (99)	1,992 (18)	163 (19)	1,829 (18)	0.26
**Perinatal characteristics**					
Gestational age (weeks), mean (CI)	11,246 (99)	40^0/7^ (40^0/7^, 40^0/7^)	40^0/7^ (39^6/7^, 40^1/7^)	40^0/7^ (40^0/7^, 40^0/7^)	0.75
Vaginal delivery, No. (%)	11,255 (100)	9,791 (87)	734 (86)	9,057 (87)	0.45
Sex (boys), No. (%)	11,255 (100)	5,873 (52)	487 (57)	5,386 (52)	0.003
Birth weight (grams), mean (CI)	11,255 (100)	3,580 (3,570, 3,580)	3,590 (3,560, 3,620)	3,580 (3,570, 3,580)	0.37
Birth weight Z-score, mean (CI)	11,255 (100)	0.015 (-0.005, 0.034)	0.034 (-0.040, 0.108)	0.013 (-0.007, 0.034)	0.59
**Antibiotic exposure, No. (%)**					
Intrapartum antibiotic exposure	11,244 (99)	1,249 (11)	104 (12)	1,145 (11)	0.29
Neonatal empirical antibiotic treatment	11,246 (99)	592 (5)	53 (6)	539 (5)	0.006
Neonatal antibiotic treatment for infection	11,246 (99)	663 (6)	69 (8)	594 (6)	
Antibiotic treatment by 6 months of age	10,857 (96)	1,762 (16)	177 (21)	1,585 (16)	< 0.001
CRP measurements No. (%)		1,331 (12)	118 (14)	1,213 (12)	

b)					
	**Total**	**Asthma**	**No asthma**	**P**	
	*n* = 11,255	*n* = 849	*n* = 10,406		
**Maternal characteristics**					
Previous births, No. (%)	5,821 (52)	439 (52)	5,382 (52)	0.99	
Maternal prepregnancy BMI *, median (CI)	23.4 (23.3, 23.4)	23.7 (23.4, 24.1)	23.3 (23.2, 23.4)	< 0.001	
Smoking during pregnancy, No. (%)	1,992 (18)	194 (23)	1,798 (17)	< 0.001	
**Perinatal characteristics**					
Gestational age (weeks), mean (CI)	40^0/7^ (40^0/7^, 40^0/7^)	39^6/7 (^39^5/7^, 39^6/7^)	40^0/7^ (40^0/7^, 40 ^0/7^)	< 0.001	
Vaginal delivery, No. (%)	9,791 (87)	723 (85)	9,068 (87)	0.099	
Sex (boys), No. (%)	5,873 (52)	538 (63)	5,335 (51)	< 0.001	
Birth weight (grams), mean (CI)	3,580 (3,570, 3,580)	3,600 (3,570, 3,630)	3,570 (3,570, 3,580)	0.16	
Birth weight Z-score, mean (CI)	0.015 (-0.005, 0.034)	0.036 (-0.035, 0.108)	0.013 (-0.008, 0.034)	0.54	
**Antibiotic exposure, No. (%)**					
Intrapartum antibiotic exposure	1,249 (11)	112 (13)	1,137 (11)	0.042	
Neonatal empirical antibiotic treatment	592 (5)	46 (5)	546 (5)	0.069	
Neonatal antibiotic treatment for infection	663 (6)	65 (8)	598 (6)		
Antibiotic treatment by 6 months of age	1,762 (16)	198 (23)	1,564 (16)	< 0.001	
CRP measurements No. (%)	1,331 (12)	110 (13)	1,221 (12)		

c)					
	**Total**	**Inhaled corticosteroid medication**	**No inhaled corticosteroid medication**	**P**	
	*n* = 11,255	*n* = 1,876	*n* = 8,981		
**Maternal characteristics**					
Previous births, No. (%)	5,821 (52)	938 (50)	4,664 (52)	0.13	
Maternal prepregnancy BMI *, median (CI)	23.4 (23.3, 23.4)	23.6 (23.4, 23.8)	23.3 (23.2, 23.4)	< 0.001	
Smoking during pregnancy, No. (%)	1,992 (18)	326 (17)	1,620 (18)	0.49	
**Perinatal characteristics**					
Gestational age (weeks), mean (CI)	40^0/7^ (40^0/7^, 40^0/7^)	39^6/7^ (39^5/7^, 39^6/7^)	40^0/7^ (40^0/7^, 40^1/7^)	< 0.001	
Vaginal delivery, No. (%)	9,791 (87)	1,634 (87)	7,803 (87)	0.80	
Sex (boys), No. (%)	5,873 (52)	1,152 (61)	4,540 (51)	< 0.001	
Birth weight (grams), mean (CI)	3,580 (3,570, 3,580)	3,590 (3,570, 3,610)	3,570 (3,560, 3,580)	0.10	
Birth weight Z-score, mean (CI)	0.015 (-0.005, 0.034)	0.030 (-0.017, 0.077)	0.013 (-0.009, 0.034)	0.52	
**Antibiotic exposure, No. (%)**					
Intrapartum antibiotic exposure	1,249 (11)	246 (13)	962 (11)	0.003	
Neonatal empirical antibiotic treatment	592 (5)	100 (5)	472 (5)	0.050	
Neonatal antibiotic treatment for infection	663 (6)	134 (7)	510 (6)		
Antibiotic treatment by 6 months of age	1,762 (16)	451 (24)	1,311 (15)	< 0.001	
CRP measurements No. (%)	1,331 (12)	235 (13)	1,051 (12)		

Continuous data are expressed as means with 95% confidence interval, and the differences between groups were assessed using T-test. Categorical data are expressed as percentages (number) and were assessed using the Chi square test

^a^Median and Wilcoxon rank-sum test were used because of the exception of normal distribution

During the follow-up, 852 (7.6%) infants were diagnosed with atopic dermatitis, 849 (7.5%) with asthma and 1,876 (16.7%) were prescribed inhaled corticosteroid medication. Atopic dermatitis was diagnosed at the median age (IQR) of 1.6 years (0.7, 4.0) and asthma at 2.7 years (1.7, 4.6). The median age for the commencement of inhaled corticosteroid medication was 2.6 years (1.6, 5.1). There was considerable overlap with the diagnoses of atopic dermatitis and asthma and inhaled corticosteroid use (Fig. 2).

**Fig. 3:**
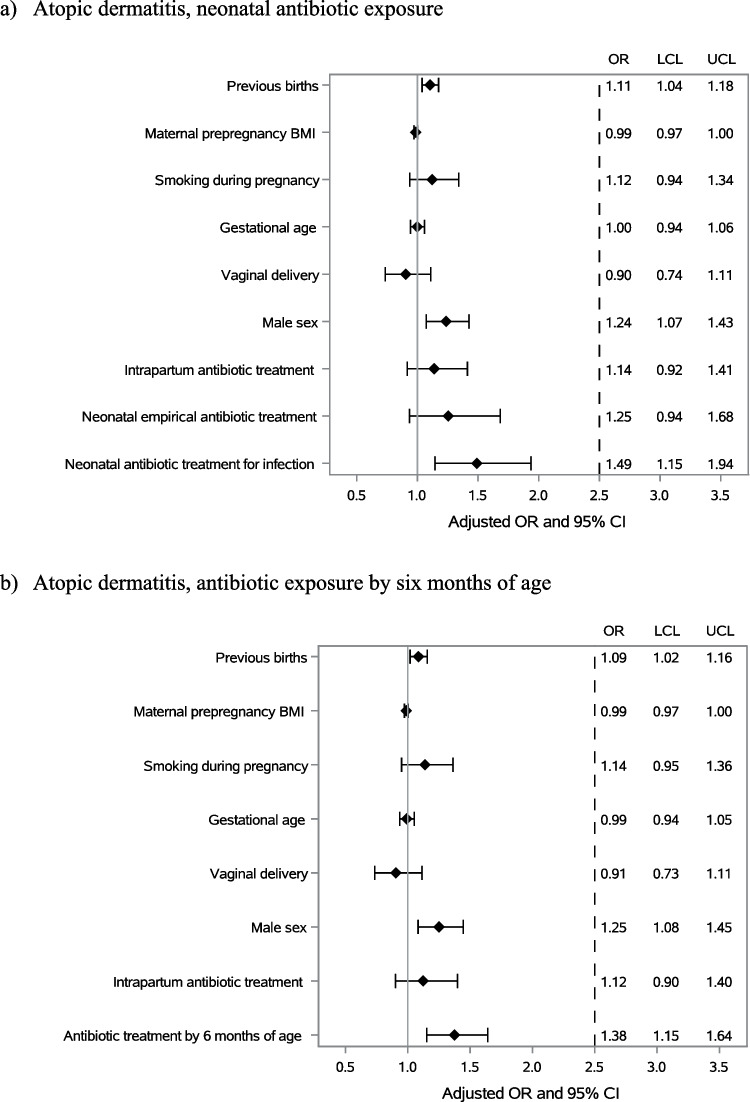

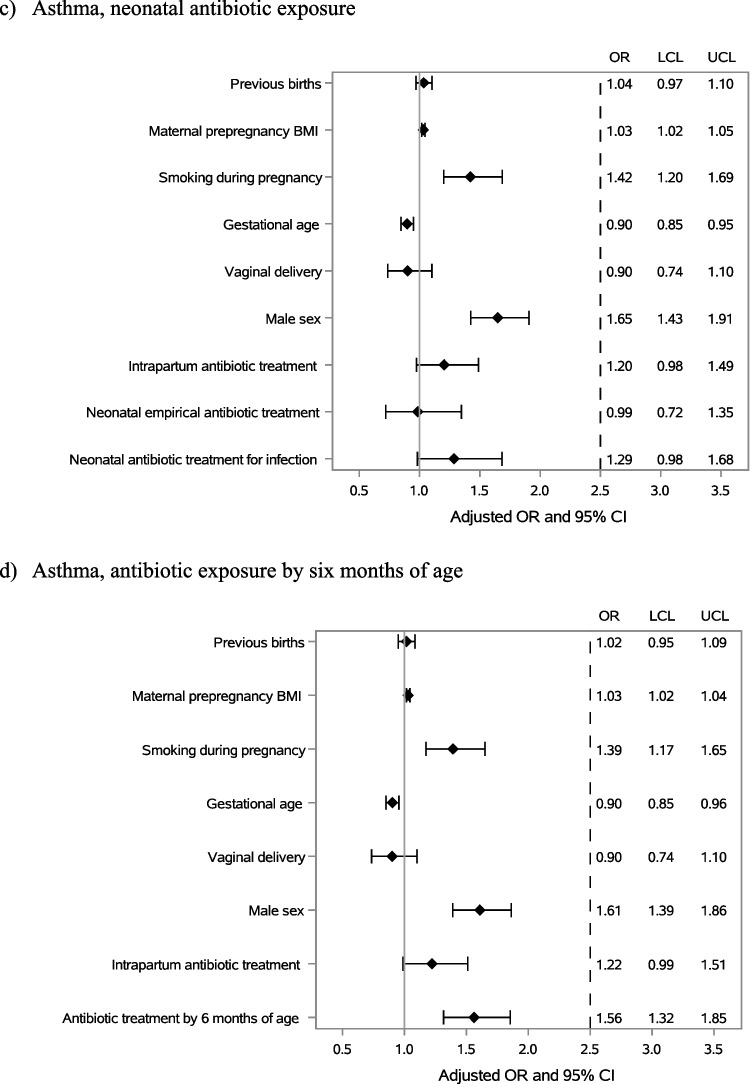

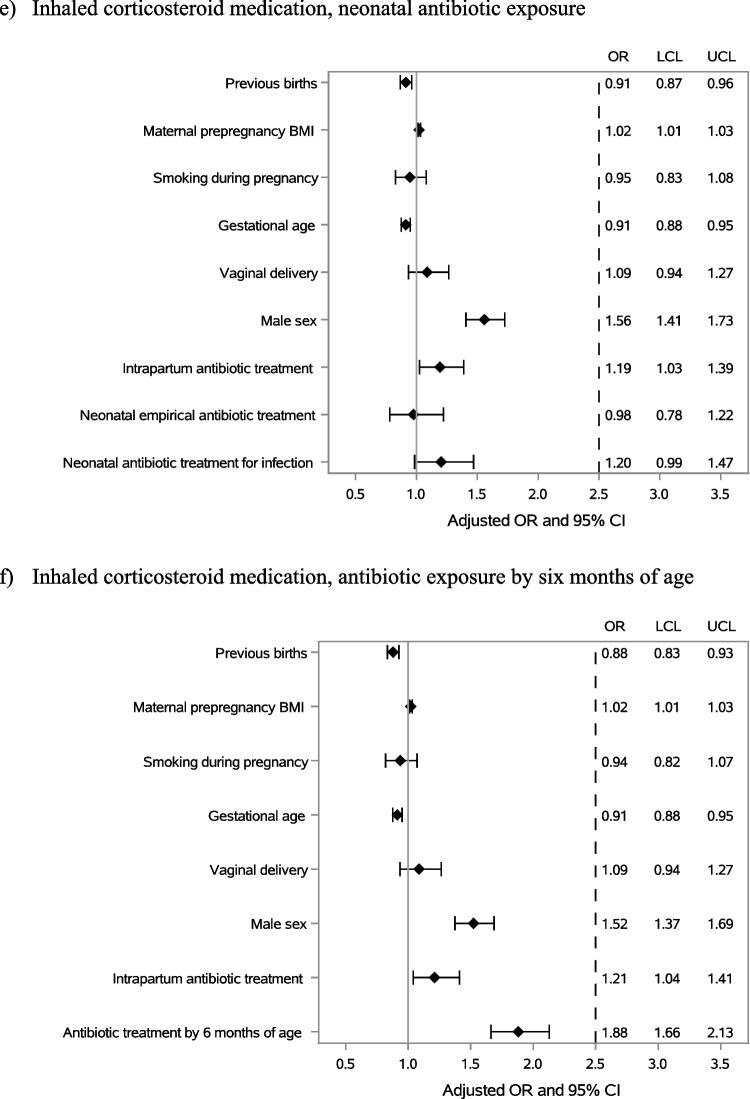
The association between antibiotic exposure in the neonatal period and the first six months of life and the development of childhood atopic dermatitis (**a**, **b**), asthma (**c**, **d**) or use of inhaled corticosteroid medication (**e**, **f**)

Antibiotic exposure both in the neonatal period and before six months of age was more common in children diagnosed with atopic dermatitis later in childhood (Table 1A). In a model adjusted for confounding factors including older siblings, maternal prepregnancy BMI, smoking during pregnancy, duration of pregnancy, mode of delivery, child’s sex and intrapartum antibiotic exposure, neonatal antibiotic exposure due to confirmed or clinically diagnosed infection was associated with the occurrence of atopic dermatitis (OR 1.49; 95% CI 1.15–1.94), whereas empirical antibiotic treatment was not (OR 1.25; 95% CI 0.94–1.68) (Fig. 3a). In a similar model, antibiotic treatment after the neonatal period but before 6 months of age was also associated with increased risk of atopic dermatitis (OR 1.38; 95% CI 1.15–1.64) (Fig. 3b).

No difference in the frequency of neonatal antibiotic exposure was detected between children who later developed asthma and those who did not (Table 1B, Fig. 3c). However, antibiotic exposure after the neonatal period but before 6 months of age was associated with higher cumulative incidence of childhood asthma during the follow-up in a model adjusted for potential confounding factors (OR 1.56; 95% CI 1.32–1.85) (Fig. 3d).

Exposure to antibiotics in the neonatal period was not associated with later use of inhaled corticosteroid medication (Table 1C, Fig. 3e). In contrast, antibiotic use after the neonatal period but before 6 months of age was more common in children who had been prescribed inhaled corticosteroid medication later in childhood (Table 1C). In models adjusted for potential confounding factors, antibiotic exposure before 6 months of age (OR 1.88; 95% CI 1.66–2.13) displayed an association with inhaled corticosteroid use (Fig. 3f).

We next examined whether the association between neonatal antibiotic exposure and later development of atopic dermatitis and use of inhaled corticosteroid use may be attributable to the inflammatory response resulting from the potentially underlying neonatal infection. Plasma concentrations of CRP had been measured from 1,331/11,255 (11.8%) study subjects during the first week of life. The maximum concentration of plasma CRP was higher in neonates with confirmed or clinically diagnosed infection as compared to those not subjected to antibiotic therapy or those with empirical antibiotic administration but in whom infection was later ruled out (Supplementary Table 2). The maximum plasma CRP concentration during the first week of life was not associated with increased risk of atopic dermatitis, asthma or inhaled corticosteroid use later in childhood (Table 2).

**Table 2:** Plasma C-reactive protein (CRP) concentrations (mg/L) in the first week of life in the children developing atopic dermatitis (a), asthma (b) or using inhaled corticosteroid medication (c) in the study cohort

a)				
**Plasma CRP** **concentration (mg/L)**	**Total**	**Atopic dermatitis**	**No atopic dermatitis**	**P**
	*n* = 1,331	*n* = 118	*n* = 1,213	
maximum, median (IQR)	4.0 (1.0, 12.0)	3.0 (1.0, 12.0)	4.0 (1.0, 12.0)	0.92
≥ 10, No. (%)	398 (30)	34 (29)	364 (30)	0.79
≥ 20, No. (%)	230 (17)	22 (19)	208 (17)	0.68
≥ 40, No. (%)	101 (8)	10 (8)	91 (8)	0.70

b)				
**Plasma CRP** **concentration (mg/L)**	**Total**	**Asthma**	**No asthma**	**P**
	*n* = 1,331	*n* = 110	*n* = 1,221	
maximum, median (IQR)	4.0 (1.0, 12.0)	4.5 (1.0, 11.0)	4.0 (1.0, 12.0)	0.92
≥ 10, No. (%)	398 (30)	33 (30)	365 (30)	0.98
≥ 20, No. (%)	230 (17)	18 (16)	212 (17)	0.79
≥ 40, No. (%)	101 (8)	5 (5)	96 (8)	0.21

c)				
**Plasma CRP** **concentration (mg/L)**	**Total**	**Inhaled corticosteroid medication**	**No inhaled corticosteroid medication**	**P**
	*n* = 1,331	*n* = 235	*n* = 1,096	
maximum, median (IQR)	4.0 (1.0, 12.0)	4.0 (1.0, 10.0)	4.0 (1.0, 13.0)	0.30
≥ 10, No. (%)	398 (30)	63 (27)	323 (31)	0.24
≥ 20, No. (%)	230 (17)	37 (16)	186 (18)	0.47
≥ 40, No. (%)	101 (8)	10 (4)	89 (8)	0.029

Continuous data are expressed as median with IQR (Q1, Q3), and the differences between groups were assessed using Wilcoxon rank-sum test. Categorical data are expressed as percentages (number) and were assessed using the Chi square test

Finally we examined the association between antibiotic exposure during the neonatal period and before 6 months of age and the composite outcome of asthma and inhaled corticosteroid use. A total of 769/11,255 (6.8%) children were both diagnosed with asthma and had a prescription of inhaled corticosteroid medication, whereas 8,901/11,255 (79.1%) subjects had neither the diagnosis, nor used inhaled corticosteroid medication. Exposure to antibiotics both in the neonatal period and before 6 months of age were more common in children who developed asthma and were prescribed with inhaled corticosteroid medication (Supplementary Table 3).

## Discussion

Our findings demonstrate that antibiotic exposure in the neonatal period and early infancy is associated with increased risk of childhood atopic disease in a large, unselected, population-based cohort. Neonatal antibiotic therapy for confirmed or clinically diagnosed infection was more common in children developing atopic dermatitis. Antibiotic treatment after the neonatal period but before six months of age was associated with the development of atopic dermatitis, asthma and inhaled corticosteroid use.

Our results are consistent with reports indicating increased risk of asthma in children treated with antibiotics during the first months or years of life [7, 13, 14]. However, it is not clear whether early antibiotic use is merely an indicator of frequent and more severe respiratory tract infections associated with asthma development. Moreover, respiratory tract infections have been proposed to play a causal role in the development of wheezing and asthma [15, 16]. We concentrated on antibiotic exposure during the first 6 months of life and preceding the onset of atopic dermatitis, asthma or inhaled corticosteroid use in our study population. To further rule out the possibility of reverse causality or confounding by viral respiratory tract infections, we separately examined antibiotic therapy for suspected or confirmed systemic bacterial infections in the first two weeks of life, which are not known to be implicated in the pathogenesis of atopic disease.

The association between neonatal antibiotic exposure and atopic dermatitis risk has not been extensively studied. Schoch and colleagues have reported [17] that antibiotic exposure during the first four weeks of life was associated with reduced occurrence of atopic dermatitis in a register-based retrospective study of more than 4000 children. In contrast to these data, we found neonatal antibiotic therapy for confirmed or clinically diagnosed bacterial infection to be associated with higher risk of later developing atopic dermatitis. The differences between the results of these two studies may be explained by the fact that we concentrated on intravenous antibiotics administered in the neonatal unit during first days of life, whereas Schoch et al. also included outpatient antibiotic prescriptions. Moreover, the cohort studied by Schoch et al. included preterm neonates, who probably were overrepresented in the population receiving antibiotics. Individuals born preterm reportedly exhibit lower risk of developing atopic dermatitis [18].

In the present study, neonatal antibiotic therapy for confirmed or clinically diagnosed bacterial infection was associated with higher risk of atopic dermatitis whereas brief antibiotic exposure without infection was not. The difference between the exposure groups may be explained by either the concomitant infection and inflammatory response or the duration of antibiotic therapy. A large proportion of the neonates diagnosed with neonatal infection exhibited little or no signs of systemic inflammatory activation as assessed by plasma CRP concentrations. Moreover, neonatal systemic inflammation was not consistently associated with the development of atopic manifestations. These data suggest that the duration of neonatal antibiotic exposure may play a more important role in the development of atopic disease than the underlying infection and inflammatory response.

Antibiotic use in the first week of life has previously been reported to be associated with increased prevalence of asthma at school age in a cohort of more than 5,000 children from Sweden [11, 19]. In the present study, we found no association between neonatal antibiotic administration, either empirical or for suspected or confirmed infection, and later asthma or inhaled corticosteroid use. The discrepant results may at least in part be explained by the longer follow-up until 12 years of age in the Swedish study, which on the other hand relied on questionnaires and might therefore suffer from recall bias.

Antibiotic exposure in the neonatal period and early infancy is reportedly associated with perturbations of the intestinal and nasal microbiome [3, 10, 20]. Given the established links between altered early gut and nasal microbiome and the risk of developing atopic dermatitis and asthma [8, 21–23], the associations between early antibiotic exposure and atopic manifestations in the present cohort may be mediated by perturbations of the developing intestinal or airway microbiome. In line with this notion, experimental studies have corroborated the causal links between early antibiotic exposure, gut microbiota perturbations and the risk of asthma [24, 25]. A causal chain of early antibiotic exposure leading to altered gut colonization which in turn may result in shifts in immune pathways [26–28] may thus contribute to the development of atopic dermatitis and asthma.

Our study has several strengths which improve the reliability and generalizability of the results. The study is based on a large, unselected birth cohort of all singleton full-term children born in a geographical area during a period of three years and, consequently, free of selection bias improving the generalizability of our results. Previous studies assessing the link between early antibiotic exposure and atopic disease have variably taken into consideration potential confounding factors such as maternal smoking and older siblings and many have relied on questionnaires or parental reports when recording outcomes [4–6, 11, 13]. In the present study, the exposure and outcome data are based on hospital and national electronic registries and are therefore free of recall bias. The diagnoses recorded in the national register reflect doctor-diagnosed conditions. In Finland, antibiotics or inhaled corticosteroids are only available by prescription by a licensed physician and the national register regarding purchases accurately captures prescriptions from all public and private clinics. Using the same data sources, we were able to take into consideration potential confounding factors including older siblings, maternal prepregnancy BMI, smoking during pregnancy, duration of pregnancy, delivery mode, child’s sex and intrapartum antibiotic exposure**.** However, data for all potential sources of confounding such as family history of atopic disease, socioeconomic background, breastfeeding or paternal smoking were not available. It is plausible that the observed associations might at least partially reflect other underlying correlations, such as the presence of abnormal symptoms during the neonatal period or factors related to treatment in a neonatal unit including but not limited to respiratory support, reduced parental interaction and potentially delayed or unsuccessful breastfeeding in the group receiving empirical antibiotic treatment. However, we lack data to support these potential associations, and there is no sound evidence linking atopic dermatitis or asthma to maternal separation.

Our results indicate that neonatal antibiotic exposure for clinically diagnosed or confirmed infection is associated with increased occurrence of atopic dermatitis and antibiotic use after the neonatal period but during the first six months of life is associated with increased risk of atopic dermatitis, asthma and inhaled corticosteroid use later in childhood. Translational research is needed to elucidate the potential causal mechanisms underlying these associations. It is important to bear in mind that antibiotic therapy is vitally important in the care of sick neonates and infants, but we must be cognizant of its potentially harmful long-term effects. Improved means of accurately identifying neonates and infants who need antibiotic therapy and novel interventions to minimize the risks in exposed children are needed.

## Electronic supplementary material

Below is the link to the electronic supplementary material.

Supplementary material 1 (DOCX 20 KB)

Supplementary material 2 (DOCX 16 KB)

Supplementary material 3 (DOCX 14 KB)

## Data Availability

No datasets were generated or analysed during the current study.

## List of abbreviations

BMI: Body mass index
CI: Confidence Interval
CRP: C-reactive protein
IQR: Interquartile Range
LCL: Lower confidence limit
UCL: Upper confidence limit
OR: Odds Ratio
SFBC: Southwest Finland Birth Cohort
