# Supplementary material for: Neonatal and early infancy antibiotic exposure is associated with childhood atopic dermatitis, wheeze and asthma
Source: Eur J Pediatr. 2024 Sep 28;183(12):5191–202. doi: 10.1007/s00431-024-05775-1 (PMC11527921; doi:10.1007/s00431-024-05775-1)
Supplement: Supplementary file 1 — Supplementary material 1 (DOCX 20 KB) [file 431_2024_5775_MOESM1_ESM.docx]

**Supplementary Table 1.** Clinical characteristics of the children exposed and not exposed to antibiotics in the neonatal period (a) or after the neonatal period but during the first six months of life (b).

a)

|  | **Total** | **No neonatal**  **antibiotics** | **Empirical neonatal**  **antibiotics** | **Antibiotics for confirmed**  **or clinical infection** | **P** |
| --- | --- | --- | --- | --- | --- |
|  | n=11,255 | n=9,991 | n=592 | n=663 |  |
| **Maternal characteristics** |  |  |  |  |  |
| Previous births, No. (%) | 5,821 (52) | 5,270 (53) | 267 (45) | 280 (42) | <0.001 |
| Maternal prepregnancy BMI *,  median (CI) | 23.8 (23.3, 23.4) | 23.3 (23.2, 23.4) | 23.9 (23.5, 24.2) | 23.8 (23.3, 24.2) | <0.001 |
| Smoking during pregnancy, No. (%) | 1,992 (18) | 1,755 (18) | 108 (18) | 128 (19) | 0.48 |
| **Perinatal characteristics** |  |  |  |  |  |
| Gestational age (weeks),  mean (CI) | 40^0/7^ (40^0/7^, 40^0/7^) | 40^0/7^ (40^0/7^, 40^0/7^) | 40^0/7^ (39^6/7^, 40^1/7^) | 40^0/7^ (39^6/7^, 40^1/7^) | 0.43 |
| Vaginal delivery, No. (%) | 9,791 (87) | 8,748 (88) | 472 (80) | 564 (85) | <0.001 |
| Sex (boys), No. (%) | 5,873 (52) | 5,140 (51) | 344 (58) | 385 (58) | <0.001 |
| Birth weight (grams),  mean (CI) | 3,580 (3,570, 3,580) | 3,570 (3,560, 3,580) | 3,590 (3,540, 3,630) | 3,680 (3,650, 3,720) | <0.001 |
| Birth weight Z-score,  mean (CI) | 0.015 (-0.005, 0.034) | 0.0002 (-0.020, 0.020) | 0.018 (-0.089, 0.124) | 0.244 (0.156, 0.332) | <0.001 |
| **Antibiotic exposure, No. (%)** |  |  |  |  |  |
| Intrapartum antibiotic exposure | 1,249 (11) | 1,057 (11) | 79 (13) | 113 (17) | <0.001 |
| Antibiotic treatment by 6 months of age | 1,762 (16) | 1,537 (16) | 118 (20) | 107 (17) | 0.013 |
| CRP measurements No. (%) | 1,331 (12) | 417 (4) | 398 (67) | 516 (78) |  |

b)

|  | **Total** | **No antibiotics during**  **the first 6 months of life** | **Antibiotic exposure during**  **the first 6 months of life** | **P** |
| --- | --- | --- | --- | --- |
|  | n=11,255 | n=8,929 | n=1,777 |  |
| **Maternal characteristics** |  |  |  |  |
| Previous births, No. (%) | 5,821 (52) | 4,296 (48) | 1,237 (70) | <0.001 |
| Maternal prepregnancy BMI *,  median (CI) | 23.8 (23.3, 23.4) | 23.3 (23.2, 23.4) | 23.8, (23.5, 24.0) | <0.001 |
| Smoking during pregnancy, No. (%) | 1,992 (18) | 1,580 (18) | 340 (19) | 0.15 |
| **Perinatal characteristics** |  |  |  |  |
| Gestational age (weeks),  mean (CI) | 40^0/7^ (40^0/7^, 40^0/7^) | 40^0/7^ (40^0/7^, 40^0/7^) | 40^0/7^ (39^6/7^, 40^0/7^) | 0.22 |
| Vaginal delivery, No. (%) | 9,791 (87) | 7,751 (87) | 1,549 (87) | 0.68 |
| Sex (boys), No. (%) | 5,873 (52) | 4,568 (51) | 1,043 (59) | <0.001 |
| Birth weight (grams),  mean (CI) | 3,580 (3,570, 3,580) | 3,560 (3,550, 3,570) | 3,640 (3,620, 3,660) | <0.001 |
| Birth weight Z-score,  mean (CI) | 0.015 (-0.005, 0.034) | -0.009 (-0.031, 0.013) | 0.149 (0.099, 0.200) | <0.001 |
| **Antibiotic exposure, No. (%)** |  |  |  |  |
| Intrapartum antibiotic exposure | 1,249 (11) | 1,028 (11) | 180 (10) | 0.18 |
| Neonatal empirical antibiotic treatment | 592 (5) | 454 (5) | 118 (7) | 0.013 |
| Neonatal antibiotic treatment for infection | 663 (6) | 537 (6) | 107 (6) |  |

Continuous data are expressed as means with 95% confidence interval, and the differences between groups were assessed using one-way Anova and T-test. Categorical data are expressed as percentages (number) and were assessed using the Chi square test. * Kruskal-wallis and Wilcoxon rank-sum test were used because of the exception of normal distribution.
