# Supplementary material for: Neonatal and early infancy antibiotic exposure is associated with childhood atopic dermatitis, wheeze and asthma
Source: Eur J Pediatr. 2024 Sep 28;183(12):5191–202. doi: 10.1007/s00431-024-05775-1 (PMC11527921; doi:10.1007/s00431-024-05775-1)
Supplement: Supplementary file 2 — Supplementary material 2 (DOCX 16 KB) [file 431_2024_5775_MOESM2_ESM.docx]

**Supplementary Table 3.** Clinical characteristics of the children developing asthma and using inhaled corticosteroid medication in the study cohort.

|  | **Asthma and Inhaled corticosteroid medication** | **No asthma and No inhaled corticosteroid medication** | **P** |
| --- | --- | --- | --- |
|  | n=769 | n=8,901 |  |
| **Maternal characteristics** |  |  |  |
| Previous births, No. (%) | 395 (51) | 4,620 (52) | 0.77 |
| Maternal prepregnancy BMI *,  median (CI) | 23.8 (23.4, 24.2) | 23.3 (23.2, 23.4) | <0.001 |
| Smoking during pregnancy,  No. (%) | 180 (23) | 1,606 (18) | <0.001 |
| **Perinatal characteristics** |  |  |  |
| Gestational age (weeks),  mean (CI) | 39 ^6/7^ (39 ^5/7^, 39 ^6/7^) | 40 ^0/7^ (40 ^0/7^, 40 ^1/7^) | <0.001 |
| Vaginal delivery, No. (%) | 647 (84) | 7,727 (87) | 0.037 |
| Sex (boys), No. (%) | 492 (64) | 4,494 (50) | <0.001 |
| Birth weight (grams),  mean (CI) | 3,600 (3,570, 3,640) | 3,570 (3,560, 3,580) | 0.078 |
| Birth weight Z-score,  mean (CI) | 0.050 (-0.026, 0.125) | 0.013 (-0.009, 0.035) | 0.36 |
| **Antibiotic exposure, No. (%)** |  |  |  |
| Intrapartum antibiotic exposure | 103 (13) | 953 (11) | 0.023 |
| Neonatal empirical antibiotic treatment | 43 (6) | 469 (5) | 0.035 |
| Neonatal antibiotic treatment for infection | 61 (8) | 506 (6) |  |
| Antibiotic treatment by 6 months of age | 190 (25) | 1,303 (15) | <0.001 |

Continuous data are expressed as means with 95% confidence interval, and the differences between groups were assessed using T-test. Categorical data are expressed as number (percentage) and were assessed using the Chi square test. *Median and Wilcoxon rank-sum test were used because of the exception of normal distribution.
