# Supplementary material for: Neonatal and early infancy antibiotic exposure is associated with childhood atopic dermatitis, wheeze and asthma
Source: Eur J Pediatr. 2024 Sep 28;183(12):5191–202. doi: 10.1007/s00431-024-05775-1 (PMC11527921; doi:10.1007/s00431-024-05775-1)
Supplement: Supplementary file 3 — Supplementary material 3 (DOCX 14 KB) [file 431_2024_5775_MOESM3_ESM.docx]

**Supplementary Table 2**. Plasma C-reactive protein (CRP) concentrations in the first week of life in the children in the study cohort.

| **Plasma CRP**  **concentration (mg/L)** | **No neonatal antibiotic treatment** | **Neonatal empirical antibiotic treatment** | **Neonatal antibiotic treatment for infection** | **P** |
| --- | --- | --- | --- | --- |
|  | n=417 | n=398 | n=516 |  |
| maximum, median (IQR) | 2.0 (0.5, 6.0) | 3.0 (1.0, 7.0) | 12.0 (3.0, 30.5) | <0.0001 |
| ≥10, No. (%) | 43 (10) | 73 (18) | 282 (55) | <0.0001 |
| ≥20, No. (%) | 12 (3) | 23 (6) | 195 (38) | <0.0001 |
| ≥40*, No (%) | 1 (0) | 7 (2) | 93 (18) | <0.0001 |

Continuous data are expressed as median with IQR (Q1, Q3), and the differences between groups were assessed using Kruskal-Wallis test. Categorical data are expressed as percentages (number) and were assessed using the Chi square test. * Fisher’s exact test was used because limited number of subjects.
